# Placental Insertion into the Cervix with Cervical Shortening as a Clinical Sign to Suspect Cervico-Isthmic Pregnancy: A Case Report and Literature Review

**DOI:** 10.1155/2023/1816955

**Published:** 2023-02-06

**Authors:** Chisa Ito, Hirotada Suzuki, Yusuke Amano, Shigeyoshi Kijima, Akihide Ohkuchi, Hironori Takahashi, Hiroyuki Fujiwara

**Affiliations:** ^1^Department of Obstetrics and Gynecology, Jichi Medical University, 3311-1 Yakushiji, Shimotsuke, Tochigi 329-0498, Japan; ^2^Department of Pathology, Jichi Medical University, 3311-1 Yakushiji, Shimotsuke, Tochigi 329-0498, Japan; ^3^Department of Radiology, Jichi Medical University, 3311-1 Yakushiji, Shimotsuke, Tochigi 329-0498, Japan

## Abstract

The clinical signs of cervico-isthmic pregnancy during pregnancy remain unknown. We herein report a case of cervico-isthmic pregnancy showing placental insertion into the cervix with cervical shortening, with a final diagnosis of placenta increta at the uterine body and cervix. A 33-year-old multiparous woman with a history of cesarean section was referred to our hospital at 7 weeks of gestation with suspected cesarean scar pregnancy. Cervical shortening with a cervical length of 14 mm was noted at 13 weeks of gestation. The placenta is gradually inserted into the cervix. An ultrasonographic examination and magnetic resonance imaging strongly suggested placenta accreta. We planned elective cesarean hysterectomy at 34 weeks of gestation. The pathological diagnosis was cervico-isthmic pregnancy with placenta increta at the uterine body and cervix. In conclusion, placental insertion into the cervix with cervical shortening in the early pregnancy period may be a clinical sign to suspect cervico-isthmic pregnancy.

## 1. Introduction

Cervico-isthmic pregnancy is defined as a placenta that partially extends beyond the histological internal cervical os. Its incidence ranges between 1 in 1,000 to 95,000 pregnancies [1, 2]. Cervico-isthmic pregnancy is often accompanied by placenta accreta, causing massive bleeding from the detached surface of the placenta at delivery, and most cases require hysterectomy [3]. However, due to its rarity, the characteristics and method of diagnosis of cervico-isthmic pregnancy have not yet been established. It is not generally diagnosed during pregnancy and is only ultimately revealed by a postoperative histopathological examination of the uterus [4]. Therefore, the clinical signs to suspect cervico-isthmic pregnancy in the mid-trimester remain unknown.

We herein report a case of cervico-isthmic pregnancy showing placental insertion into the cervix with cervical shortening, with a final diagnosis of placenta increta at the uterine body and cervix, and reviewed case reports and case series of cervico-isthmic pregnancy according to gestational age at delivery. We propose clinical signs to suspect cervico-isthmic pregnancy.

## 2. Case Presentation

**Figure 1:**
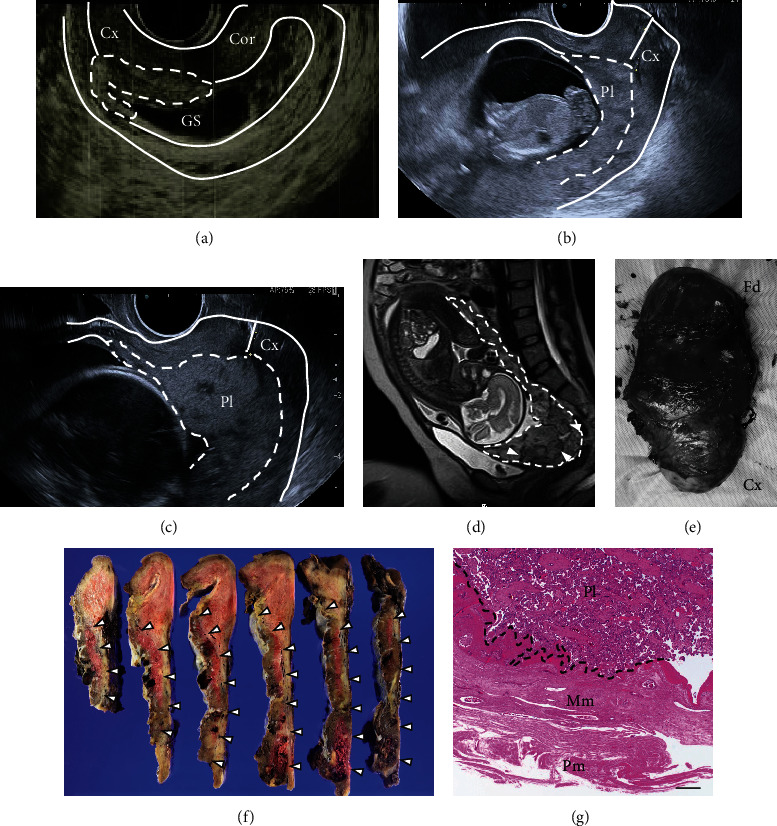
(a)–(c) Transvaginal ultrasound findings. (a) Low implantation of the gestational sac and the placenta attached to the posterior wall of the uterus (dotted line) are observed at 7 weeks of gestation. The anterior myometrium including the cesarean scar is intact. (b) The cervical length is 14 mm at 13 weeks of gestation. (c) Placental insertion into the cervix and cervical shortening are noted at 20 weeks of gestation. (d) Magnetic resonance imaging findings at 30 weeks of gestation. Sagittal T2-weighted half Fourier single-shot turbo spin echo (HASTE) sequence images show the insertion of the placenta into the effacement of the cervical canal, intraplacental vasodilatation (white arrowheads), and heterogeneity within the placenta. (e) Surgical specimen of cesarean hysterectomy. A swollen lower uterine segment is noted. (f) and (g) Histopathological finding. (f) White arrowheads indicate the boundaries of the placenta and myometrium. The placenta extensively adhered to the anteroposterior wall from the lower uterine segment to the cervix. (g) The placenta invading the myometrium (dotted line) is observed. The scale bar indicates 500 *μ*m. GS, gestational sac; Cx, cervix; Cor, corpus of the uterus; Pl, placenta; Fd, fundus; Mm, myometrium; Pm, perimetrium.

**Figure 2:**
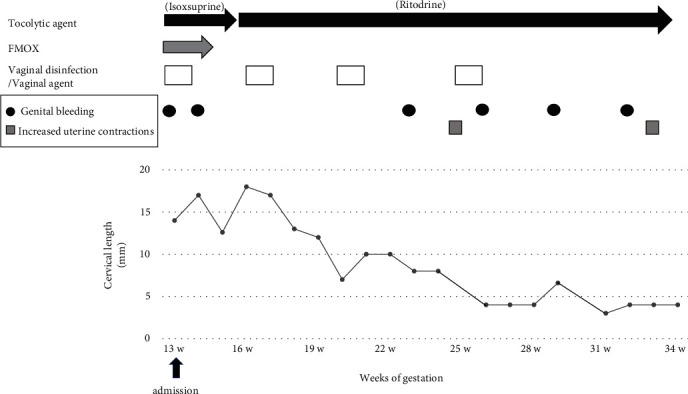
A diagram showing the clinical course of the patient. The cervical length decreased with a small amount of genital bleeding as gestation progressed. FMOX, flomoxef.

A 33-year-old multiparous woman with a previous obstetric history of cesarean section due to breech presentation naturally conceived and was referred to our hospital at 7 weeks of gestation with suspected cesarean scar pregnancy (CSP). A transvaginal ultrasonographic examination revealed low implantation of the gestational sac (GS) with a heartbeat (Figure 1(a)). However, the anterior myometrium including the cesarean scar was intact, ruling out a diagnosis of CSP. The patient strongly desired to continue the pregnancy. She was admitted to our hospital at 13 weeks of gestation with threatened miscarriage due to genital bleeding and a shortened cervical length of 14 mm. Thereafter, a small amount of genital bleeding was occasionally observed, and the placenta was gradually inserted into the cervix with cervical shortening (Figures 1(b), 1(c), and 2). Placenta accreta was strongly suspected because an ultrasonographic examination showed the disappearance of the sonolucent zone, scattered lacunae, and abundant inflow of blood vessels in the placental space from the posterior wall to the anterior wall near the internal cervical os. Magnetic resonance imaging (MRI) at 30 weeks of gestation revealed insertion of the placenta into the effacement of the cervical canal, irregularity and thinning of the myometrium, loss of the signal at the placental muscle layer boundary, intraplacental vasodilatation, intraplacental hypointense areas, and heterogeneity within the placenta (Figure 1(d)), which strongly suggested placenta accreta. We predicted the risk of massive bleeding during delivery to be very high based on ultrasonographic images and MRI findings of placenta accreta.

After the administration of betamethasone preoperatively to promote fetal lung maturation, elective cesarean hysterectomy was performed in a hybrid operating room at 34 weeks and 1 day of gestation. Under spinal anesthesia, bilateral ureteral stents were placed by a urologist to facilitate the identification of the ureters. Resuscitative endovascular balloon occlusion of the aorta (REBOA) and a catheter for uterine artery embolization (UAE) in the right internal iliac artery were then placed by an interventional radiologist to prepare for massive bleeding during and after hysterectomy. No abnormal blood vessels or findings suggestive of CSP were detected on the anterior uterine surface around the previous cesarean scar. We performed transverse hysterotomy at the fundus of the uterus and delivered a male infant weighting 1,938 g with Apgar scores of 8/9 (1/5 minutes). We then switched to general anesthesia, followed by cesarean hysterectomy after suturing transverse hysterotomy without detaching the placenta. Regarding the incision of the previous cesarean section, the anterior wall placenta was not placenta percreta, but placenta increta; therefore, there was no bladder invasion, and the bladder was smoothly detached. The volume of bleeding was not high before the vaginal stump procedure. Since massive bleeding was expected during resection of the vaginal stump to suturing during hysterectomy, REBOA was inflated for 6 minutes from resection of the vaginal stump to suturing, and the uterus was completely removed without massive bleeding. Intraoperative blood loss was 1,770 g. Six units of red blood cells were transfused because hemoglobin decreased to 7 mg/dL with continuous bleeding during hysterectomy. The patient was transferred to the intensive care unit for close observations of the abrupt occurrence of postoperative intra-abdominal bleeding. On the next day, she was returned to the obstetrics ward and was discharged with no complications on the 8th postoperative day.

Upon gross inspection, the lower uterine segment was dilated due to the placenta, the cervix had effaced (Figure 1(e)), and the placenta was visible from the cervical os. A histopathological examination of the placenta and uterus revealed placenta previa with extensive adhesions into the anteroposterior wall, mainly into the posterior wall from the lower uterine segment to the cervix (Figures 1(f) and 1(g)). The final histopathological diagnosis was cervico-isthmic pregnancy with placenta increta at the uterine body and cervix.

## 3. Discussion

We propose placental insertion into the cervix with cervical shortening in the early second trimester as a clinical sign to suspect cervico-isthmic pregnancy.

We reviewed case reports and case series of cervico-isthmic pregnancy according to gestational age at delivery. PubMed/Medline and Igaku Chuo Zasshi (Ichushi) for a bibliographic database of medical literature published in Japan were used to search for articles that described cervico-isthmic pregnancy, with articles in English and Japanese both being included. The following terms were used: “cervico-isthmic pregnancy”, “cervical pregnancy with placenta accreta”, “cervical placenta”, and “placenta increta cervicalis”. The last update of the search was May 31, 2022. Reviews and articles without detailed data descriptions were excluded. The article search identified 23 articles (23 patients) in PubMed/Medline and 10 (13 patients) in Ichushi. Thirty-seven patients with cervico-isthmic pregnancy, including the present case, were analyzed according to gestational age at delivery: ≤11 weeks of gestation (*n* = 9), 12–21 weeks of gestation (*n* = 6), and ≥22 weeks of gestation (*n* = 22) (Table 1). The maternal characteristics of 37 patients with cervico-isthmic pregnancy were a high rate of patients with previous histories of uterine surgery (e.g., cesarean section or dilation and curettage) (70.3%) as well as high rates of primipara/primigravida (40.5%/24.3%) and spontaneous conception (81.1%). Regarding imaging findings, low implantation of the GS at ≤11 weeks of gestation and placental insertion into the cervix and cervical shortening at 12–21 weeks of gestation were frequently observed. At ≥22 weeks of gestation, some patients showed placental insertion into the cervix and cervical shortening. These findings strongly suggested placental insertion into the cervix with cervical shortening in the mid-trimester as a clinical sign to suspect cervico-isthmic pregnancy.

Placenta accreta frequently occurs in women with cervico-isthmic pregnancy. In our review (Table 1), 5 out of 6 women (83%) with cervico-isthmic pregnancy with termination at 12–21 weeks of gestation underwent hysterectomy. Among 22 women with cervico-isthmic pregnancy with termination at ≥22 weeks of gestation, 19 (86%) underwent hysterectomy. Therefore, if the continuation of cervico-isthmic pregnancy is desired, we need to plan cesarean hysterectomy with careful preparation for possible massive bleeding during and after hysterectomy. In dangerous cases with a high risk of placenta accreta/increta/percreta, we typically perform the setting of bilateral ureteral stents and REBOA as well as a catheter for UAE in the right internal iliac artery. In the present case, we performed cesarean hysterectomy safely and without massive bleeding during hysterectomy under the above-described preparation.

Cervical pregnancy generally implants in the cervix only, and abnormal blood vessels dilate and meander, resulting in the swelling of the cervical canal. As the GS enlarges, it causes heavy bleeding, making it difficult to continue the pregnancy [5, 6]. Cervico-isthmic pregnancy is considered to implant across the cervical canal and cervico-isthmus of the uterus between the histological and anatomical internal os [1, 7]. In the present case, we detected the gradual insertion of the placenta into the cervix and cervical shortening with the progression of gestation. In addition, since the amniotic fluid cavity extends towards the fundus of the uterus, pregnancy may continue in some cases. Placenta previa generally implants in the corpus and not the cervico-isthmus of the uterus; therefore, it does not generally cause placenta accreta at the uterine cervix. It may be difficult to distinguish between cervico-isthmic pregnancy with cervical shortening and placenta previa with cervical shortening due to threatened premature delivery. Since the clinical course, findings, and treatments differ depending on the site (cervix, cervico-isthmus, and corpus of the uterus) at which the GS implants, it is important to pay attention to the implantation site in the first trimester and carefully observe the course of the pregnancy.

Cervico-isthmic pregnancy maybe prone to placenta accreta with extensive adhesions to the anterior and posterior walls, as in the present case. CSP is similar to cervico-isthmic pregnancy. CSP implants in a microscopic or macroscopic tract on the scar or in the niche (triangular defect) at the site of a previous cesarean section [8, 9]. The basic incision site for cesarean section is the anterior lower uterine segment, which is the extension of the cervico-isthmus of the uterus. Since the endometrium of the cervico-isthmus is thinner than that of the corpus, the decidual change is weak, and a complete compact layer cannot be formed. A cesarean scar has a thin or absent decidua around the isthmus of the uterus; therefore, CSP may be prone to placenta accreta mainly on the anterior wall, which may lead to bladder infiltration and even rupture. Cervico-isthmic pregnancy implants in the isthmus of the uterus, at which the endometrium is circumferentially thin, and, thus, may be prone to placenta accreta on the anterior and posterior walls.

It is clinically beneficial to suspect cervico-isthmic pregnancy as early as possible. Not all obstetricians recognize and diagnose cervico-isthmic pregnancy due to its rarity, and total hysterectomy may be required at the time of delivery after 12 weeks of gestation. Inadvertent induced abortion or delivery management without cervico-isthmic pregnancy being suspected may lead to a delayed response to massive bleeding and contribute to acceptance by a pregnant woman of the unintended outcome of total hysterectomy. Cervico-isthmic pregnancy may be suspected if the GS is present in the lower part of the uterus in early pregnancy, and based on this finding, a pregnant woman may be informed that she has a high-risk pregnancy that may require total hysterectomy. However, difficulties are associated with identifying the implantation site of the GS in pregnant women in their first visit after the second trimester of pregnancy. The finding of placental insertion into the cervix with cervical shortening in the mid-trimester may lead to cervico-isthmic pregnancy being suspected in pregnant women with unknown implantation sites. Furthermore, the diagnosis of cervico-isthmic pregnancy may be facilitated by combining ultrasound and MRI images, which may contribute to the policy of treatment selection by providing appropriate information to pregnant women. Therefore, placental insertion into the cervix with cervical shortening in the mid-trimester as a clinical sign to suspect cervico-isthmic pregnancy is an important finding.

Although case reports of cervico-isthmic pregnancy have been reviewed [4, 10], they have not been summarized according to gestational age at delivery or characterized, as in this review. The number of case reports of cervico-isthmic pregnancy is still limited. In this review, it was unclear how far below the cervico-isthmus of the uterus the placenta must implant to cause placental insertion into the cervix with cervical shortening, how cervico-isthmic pregnancy develops, and what types of cases may continue pregnancy. Therefore, the further accumulation of cases is needed to elucidate the etiology of cervico-isthmic pregnancy and predict its outcome.

In conclusion, we propose placental insertion into the cervix with cervical shortening in the early second trimester as a clinical sign to suspect cervico-isthmic pregnancy. It is very important to pay attention to the implantation site in the first trimester and carefully observe the course of the pregnancy. Moreover, the further accumulation of cases is needed to elucidate the pathophysiology of cervico-isthmic pregnancy.

## Figures and Tables

**Table 1:** Clinical characteristics of cervico-isthmic pregnancy according to gestational age at delivery.

	≤11 weeks of gestation (*n* = 9)		12–21 weeks of gestation (*n* = 6)		≥22 weeks of gestation (*n* = 22)	
Gestational weeks at delivery (quartile)	8 (6–9)		17 (16–18)		35.5 (33.25–37)	
Imaging findings		*n*		*n*		*n*
	Low implantation of GS	7	Placental insertion into the cervix	3	Low implantation of the gestational sac	5
			Cervical shortening	2	Placental insertion into the cervix	5
					Cervical shortening	6
					Abnormal vascularization	4
					Placental lacunae	4
					Obscurity in placenta-myometrial interface	4
Treatment or delivery method		*n*		*n*		*n*
	MTX	2	MTX → UAE → evacuation by placental forceps	1	Emergency C/S → hysterectomy	7
	MTX → UAE → TCR	2	D&E → evacuation by placental forceps → hysterectomy	1	Caesarean hysterectomy	6
	Misoprostol → D&E → hysterectomy	2	Induced delivery → D&E	1	Elective C/S → hysterectomy	3
	MTX → partial resection by laparotomy	1	Induced delivery → UAE → hysterectomy	1	Vaginal delivery → hysterectomy	3
	Aspiration of GS	1	Induced delivery → hysterectomy	1	Emergency C/S → partial resection	1
	Hysterectomy	1	Hysterectomy	1	Emergency C/S → manual removal	1
					Elective C/S → MTX → UAE → evacuation by placental forceps	1
Blood loss						mL (quartile)
					Total	3,865 (2,880–5,512)
					Elective hysterectomy	3,233 (2,481–4,203)
					Emergency hysterectomy	4,280 (3,500–7,000)
					Others∗	2,750 (2,125–3,375)

An article search identified 23 articles (23 patients) in PubMed/Medline and 10 (13 patients) in Ichushi. ∗: Manual removal of the placenta or partial resection of the lower uterine segment.

## Data Availability

The reference data supporting this review are from previously reported studies and datasets, which have been cited. The processed data are available from the corresponding author upon request.
